# Association between non-high-density lipoprotein to high-density lipoprotein ratio and reversion to normoglycemia in people with impaired fasting glucose: a 5-year retrospective cohort study

**DOI:** 10.1186/s13098-023-01237-0

**Published:** 2023-12-17

**Authors:** Zihe Mo, Yong Han, Changchun Cao, Qingli Huang, Yanhua Hu, Zhiqun Yu, Haofei Hu

**Affiliations:** 1 Department of Physical Examination, Dongguan Tungwah Hospital, No. 1 Dongcheng Road, Dongcheng Street, Dongguan, 523000 Guangdong China; 2 Department of Emergency, Shenzhen Second People’s Hospital, Shenzhen, 518000 Guangdong China; 3 Department of Emergency, The First Affiliated Hospital of Shenzhen University, Shenzhen, 518000 Guangdong China; 4 Department of Rehabilitation, Shenzhen Dapeng New District Nan’ao People’s Hospital, Shenzhen, 518000 Guangdong China; 5 College of Information Science and Engineering, Liuzhou Institute of Technology, No. 99, Xinliu Avenue, Yufeng District, Liuzhou, 545616 Guangxi Zhuang Autonomous Region China; 6 Department of Nephrology, Shenzhen Second People’s Hospital, No.3002 Sungang Road, Futian District, Shenzhen, 518000 Guangdong China; 7 Department of Nephrology, The First Affiliated Hospital of Shenzhen University, Shenzhen, 518000 Guangdong China

**Keywords:** Impaired fasting glucose, Regression to normoglycemia, Non-high-density lipoprotein to high-density lipoprotein ratio, Cox proportional-hazards regression model, Competitive risk model, Non-linear relationship

## Abstract

**Objective:**

The relationship between the non-high-density lipoprotein to high-density lipoprotein ratio (non-HDL-c/HDL-c ratio) and changes in glycemic status as well as the development of type 2 diabetes mellitus (T2DM) has been well established. However, there is a lack of evidence concerning the association between the non-HDL-c/HDL-c ratio and the reversal of normoglycemia in individuals with impaired fasting glucose (IFG). Therefore, this study aimed to examine the connection between the non-HDL-c/HDL-c ratio and the likelihood of reverting to normoglycemia among people with IFG.

**Methods:**

This retrospective cohort study examined data collected from 15,524 non-selective participants with IFG at the Rich Healthcare Group in China between January 2010 and 2016. The Cox proportional-hazards regression model was used to investigate the connection between the baseline non-HDL-c/HDL-c ratio and the probability of reverting to normoglycemia. We were able to discover the non-linear association between the non-HDL-c/HDL-c ratio and reversion to normoglycemia using a Cox proportional hazards regression model with cubical spline smoothing. We also performed several sensitivity and subgroup analyses. A competing risk multivariate Cox regression was utilized as well to examine the development to diabetes as a competing risk for the reversal of normoglycemic events.

**Results:**

In our study, a total of 15,524 individuals participated, with a mean age of 50.9 ± 13.5 years, and 64.7% were male. The average baseline non-HDL-c/HDL-c ratio was 2.9 ± 0.9. Over a median follow-up period of 2.9 years, we observed a reversion rate to normoglycemia of 41.8%. After adjusting for covariates, our findings revealed a negative association between the non-HDL-c/HDL-c ratio and the likelihood of reverting to normoglycemia (HR = 0.71, 95% CI 0.69–0.74). Notably, we identified a non-linear relationship between the non-HDL-c/HDL-c ratio and the probability of transitioning from IFG to normoglycemia. We found an inflection point at a non-HDL-c/HDL-c ratio of 3.1, with HRs of 0.63 (95% CI 0.69, 0.74) on the left side and 0.78 (95% CI 0.74, 0.83) on the right side of the point. Competing risks multivariate Cox's regression, sensitivity analysis, and subgroup analysis consistently supported our robust results.

**Conclusion:**

Our study has revealed a negative and non-linear relationship between the non-HDL-c/HDL-c ratio and reversion to normoglycemia in Chinese people with IFG. Specifically, when the non-HDL-c/HDL-c ratio was below 3.1, a significant and negative association with reversion to normoglycemia was observed. Furthermore, keeping the non-HDL-c/HDL-c ratio below 3.1 significantly elevated the probability of returning to normoglycemia.

**Supplementary Information:**

The online version contains supplementary material available at 10.1186/s13098-023-01237-0.

## Introduction

Diabetes has emerged as a significant global public health concern, with its prevalence reaching epidemic proportions [1]. The majority of patients experience a phase known as impaired fasting glucose (IFG) over the course of the disease, which is characterized by fasting plasma glucose (FPG) levels that are above the normal range but below the diagnostic cut-off for type 2 diabetes mellitus (T2DM) [2]. Pre-diabetes is defined in accordance with the 2022 criteria established by the American Diabetes Association, encompassing IFG (FPG level of 5.6–6.9 mmol/L), impaired glucose tolerance (IGT), and/or glycated hemoglobin A1c (HbA1c) [3]. According to the International Diabetes Federation (IDF), approximately 463 million people between the ages of 20 and 79 (6.0% of the global population) have prediabetes [4]. The progression rates to T2DM for this population range between 5 and 10% per year [5], with a lifetime risk exceeding 70%, especially among those who are overweight or obese [6]. Additionally, prediabetes is associated with an elevated risk of cardiovascular and microvascular diseases, as well as overall mortality [7–10].

However, the likelihood of developing T2DM from pre-diabetes varies based on factors such as sex, age, geographical location, ethnicity, social status, and the specific criteria used to define pre-diabetes [7]. Remarkably, between 20 and 50% of individuals with pre-diabetes experience a reversion back to normoglycemia instead of progressing to T2DM. Notably, findings from the DPPOS (Diabetes Prevention Program Outcomes Study) and the Special Diabetes Program for Indians Diabetes Prevention Program have demonstrated that individuals who successfully revert to normoglycemia have a decreased risk of developing diabetes mellitus [11, 12]. This suggests that the key to preventing diabetes and its complications lies in restoring normoglycemia rather than simply maintaining prediabetes. Moreover, the reversion from IFG to normoglycemia has been associated with improvements in various cardiovascular risk factors [13]. A recent study conducted in a Chinese population further indicated that reversion from IFG, as defined by fasting plasma glucose levels, to normoglycemia was linked to a reduced future risk of chronic cardiovascular disease (CVD) and all-cause mortality [14]. These findings provide compelling evidence that true prevention of diabetes and its complications is likely achieved through reversing IFG and restoring normal glucose regulation (NGR).

The total amount of cholesterol found in lipoproteins other than high-density lipoprotein cholesterol (HDL-c) is known as non-high-density lipoprotein cholesterol (non-HDL-c). In addition to observable impairments in glucose metabolism, individuals with diabetes frequently manifest atherosclerotic lipid irregularities, which are distinguished by heightened concentrations of non-HDL-c and triglycerides (TG), as well as diminished levels of HDL-c concentration [15, 16]. A higher risk of insulin resistance and diabetes has been linked to these lipid indicators of atherogenic lipoproteins, according to earlier investigations [17, 18]. The non-HDL-c/HDL-c ratio, a novel composite measure incorporating data on both atherogenic and anti-atherogenic lipid particles [19], has been shown in recent studies to independently evaluate the likelihood of nonalcoholic fatty liver disease (NAFLD), chronic kidney disease (CKD), and metabolic syndrome [20–22]. Furthermore, an observational investigation has indicated that the non-HDL-c/HDL-c ratio outperforms other markers in predicting metabolic syndrome and insulin resistance [22]. Additionally, several recent studies have established a close association between the non-HDL-c/HDL-c ratio and the occurrence and progression of IFG and T2DM [23–26]. Moreover, a further study found that the non-HDL-c/HDL-c ratio serves as a superior marker for predicting diabetes risk compared to conventional lipid parameters in the general population [27]. However, the current body of literature does not present any empirical support for a correlation between the non-HDL-c/HDL-c ratio and the regression of IFG to normoglycemia. Nevertheless, preliminary findings from prior epidemiological investigations indicate that regular physical activity, weight loss, adherence to a healthy diet, and the absence of hepatic steatosis are independently associated with an increased likelihood of reverting to normoglycemia in adults with initial prediabetic conditions [28, 29]. Based on the synthesis of the aforementioned studies, we posit the hypothesis that a potential negative correlation exists between the non-HDL-c/HDL-c ratio and the probability of reversing IFG to normoglycemia. In order to explore this association within the Chinese community, we conducted a cohort study to examine the relationship between the non-HDL-c/HDL-c ratio and the likelihood of IFG transitioning to normoglycemia.

## Methods

### Study design

In order to investigate the association between the non-HDL-c/HDL-c ratio and the reversion of IFG to normoglycemia, we conducted a retrospective cohort study utilizing data from the China Rich Healthcare Group database. Our analysis specifically examined the non-HDL-c/HDL-c ratio at baseline as the independent variable of interest, while considering the outcome variable of IFG reversion to normoglycemia during follow-up.

### Data source

The raw data was taken from the DATADRYAD database (www.datadryad.org) for free provided by Chen, Ying et al. (2018), Data from: Association of body mass index and age with incident diabetes in Chinese adults: a population-based cohort study, Dryad, Dataset, https://doi.org/10.5061/dryad.ft8750v. Researchers are granted permission to utilize this dataset for non-commercial purposes in accordance with the terms of service specified by the Dryad database. They are also allowed to share, modify, remix, and create derivative works based on the dataset, as long as appropriate credit is given to the author and source [30].

### Study population

To minimize selection bias, participants were consecutively enrolled from 32 locations across 11 cities in China (Suzhou, Nanjing, Changzhou, Shanghai, Beijing, Shenzhen, Guangzhou, Chengdu, Nantong, Hefei, and Wuhan) by the Rich Healthcare Group. The study employed non-traceable codes to encode participant identity information to mitigate privacy apprehensions. The data utilized in this research was extracted from a computerized database established by the Rich Healthcare Group in China, which housed medical records of individuals who underwent health examinations from 2010 to 2016. This study adhered to the principles outlined in the Declaration of Helsinki, and all procedures involving human subjects were approved by the clinical research ethics committee of the Rich Healthcare Group. The Institutional Review Board waived the necessity for informed consent from participants due to the retrospective design and anonymized nature of the data [30, 31].

**Fig. 1:**
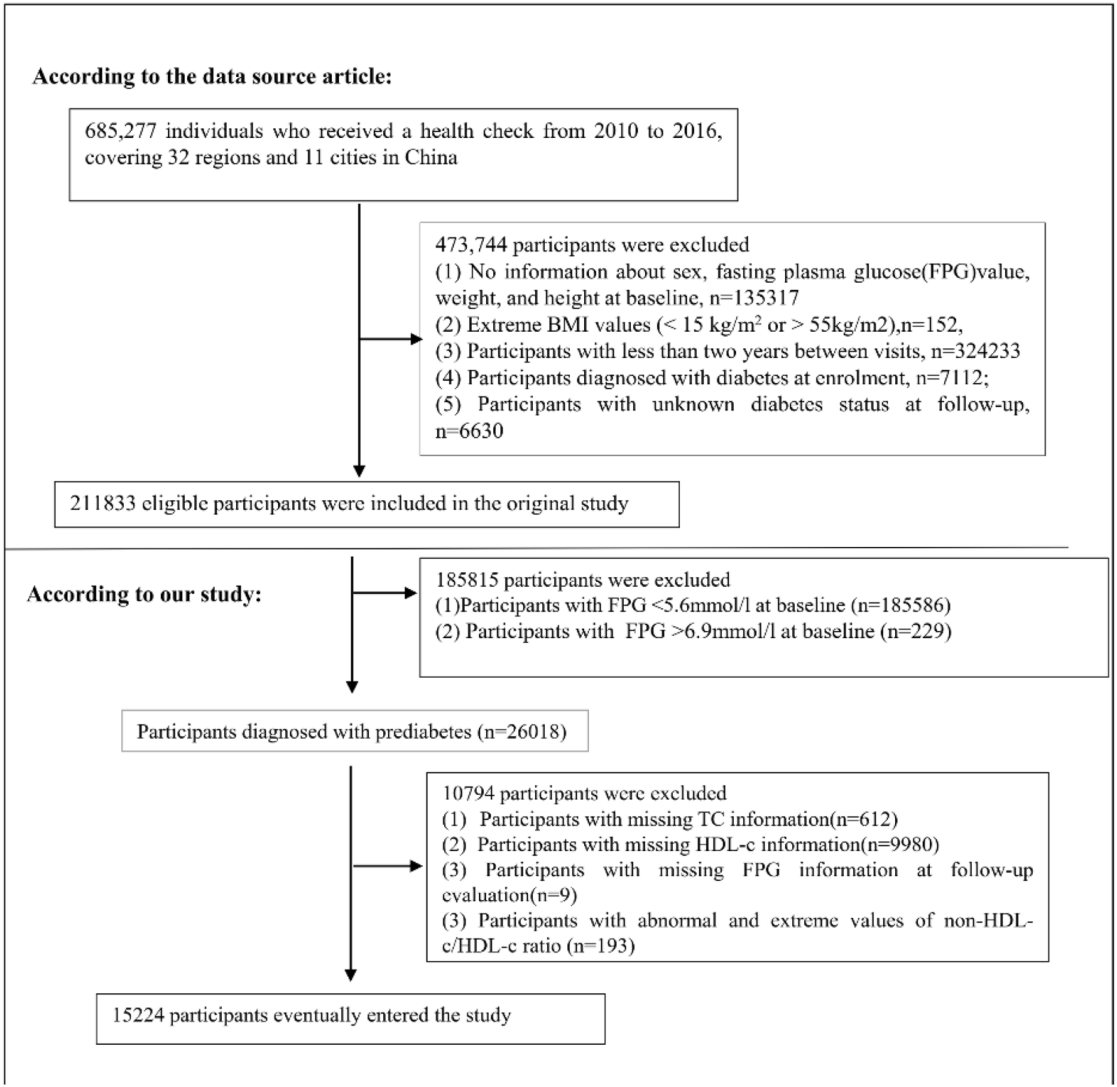
Flowchart of study participants. Illustrates the participant selection process. Initially, a total of 211,833 participants were evaluated for eligibility in the original study. After excluding 196,609 individuals, the final analysis consisted of 15,224 subjects in the current investigation

The original study initially included 685,277 participants aged 20 or above who had undergone at least two health examinations. Following the exclusion of 670,053 participants, 15,224 participants remained for data analysis (refer to Fig. 1 for a detailed flowchart). The entire study adheres to the Strobe statement. The exclusion criteria comprised the following: (1) visit period less than 2 years; (2) extreme body mass index (BMI) values (< 15 or > 55 kg/m^2^); (3) incomplete records of weight, sex, height, and FPG value at baseline; (4) pre-existing diagnosis of diabetes; and (5) unknown diabetes status at follow-up. After applying these exclusion criteria, the analysis involved a total of 211,833 individuals in the original study [30]. In the current study, we conducted further exclusions on an additional 185,815 subjects who exhibited baseline FPG levels below 5.6 mmol/L or above 6.9 mmol/L. Moreover, participants without baseline data on total cholesterol (TC) or HDL-c were excluded (n = 10,592), as well as those lacking FPG information during follow-up (n = 9), and individuals displaying extreme non-HDL-c/HDL-c ratio values that were considered anomalous (values greater or less than three standard deviations from the mean) (n = 193) [32].

### Variables

#### Non-high-density lipoprotein to high-density lipoprotein ratio

We collected the baseline information of the non-HDL-c/HDL-c ratio and recorded it as a continuous variable. The calculation procedure for obtaining the non-HDL-c/HDL-c ratio is described below. The non-HDL-c/HDL-c ratio is calculated by dividing the non-HDL-c value (measured in mmol/L) by the HDL-c value (measured in mmol/L). To determine the non-HDL-c value, the HDL-c value is subtracted from the total cholesterol (TC) measurement [33].

#### Outcome measures

The occurrence of reversion to normoglycemia, defined as having an FPG level of less than 5.6 mmol/L during the follow-up assessment and no self-reported incident diabetes, was our primary outcome of interest [3, 34].

#### Covariates

The covariates included in this study were selected based on our clinical experiences and previous research examining risk factors for IFG, diabetes, or reversion to normoglycemia [24, 26, 28–30, 35]. Following these principles, the following variables were considered as covariates: (1) continuous variables: age, FPG, BMI, blood urea nitrogen (BUN), diastolic blood pressure (DBP), serum creatinine (Scr), TG, alanine aminotransferase (ALT), systolic blood pressure (SBP), low-density lipid cholesterol (LDL-c), aspartate aminotransferase (AST), (2) categorical variables: gender, age, smoking and drinking status, family history of diabetes.

#### Data collection

During every visit to the health check center, participants were given a comprehensive questionnaire on their lifestyle, family history of diabetes, personal medical history, and demographic characteristics. Trained staff meticulously measured height, blood pressure, and weight with utmost precision and accuracy. In order to ensure precise weight measurements, participants were instructed to wear lightweight attire and refrain from wearing shoes while recording measurements to the nearest 0.1 kg. The calculation of BMI involved dividing the weight (in kilograms) by the square of the height (in meters), with height measurements taken with a precision of 0.1 cm. Participants' blood pressure was measured using standard mercury sphygmomanometers in an office setting. Prior to measurement, participants were instructed to rest quietly for 5–10 min in a lying position. Participants' smoking behavior was categorized into three groups: current smokers, former smokers, and non-smokers. Similarly, participants' drinking status was classified as current drinkers, former drinkers, or non-drinkers. Assessments of smoking and drinking status were conducted only at baseline. Venous blood samples were collected during each visit following a minimum fasting period of 10 h. These samples were later analyzed for HDL-c, BUN, TC, AST, TG, Scr, FPG, LDL-c, and ALT utilizing an autoanalyzer (Beckman 5800) [30].

#### Missing data processing

A small percentage of participants in the study had missing data for certain variables. Specifically, 5 participants (0.033%) were missing SBP and DBP data, while 28 participants (18.39%) had missing LDL-c data. Furthermore, a larger proportion of participants, namely 8188 (53.78%), 10556 (69.34%), and 10556 (69.34%), had missing data for AST, smoking status, and drinking status, respectively. Additionally, 37 participants (0.24%) were missing ALT data, 360 participants (2.36%) were missing BUN data, and 116 participants (0.76%) were missing Scr data. To maximize the utilization of participants' data and minimize potential bias resulting from missing data, we employed multiple imputation by chained equations [36]. This imputation included variables such as BMI, SBP, age, Scr, gender, DBP, ALT, BUN, FPG, TG, family history of diabetes, LDL-c, AST, and drinking and smoking status. The missing data were analyzed based on the assumption that they were missing at random (MAR) [37].

### Statistical analysis

The participants were classified into quartiles of the non-HDL-c/HDL-c ratio. Descriptive statistics were used to present continuous variables, with mean ± standard deviation (SD) for normally distributed variables and median (range) for skewed variables. Categorical variables were reported as frequencies and percentages. To assess differences among the non-HDL-c/HDL-c ratio groups, χ2 tests, One-Way ANOVA tests, and Kruskal–Wallis H tests were employed for categorical variables, normally distributed variables, and skewed variables, respectively. We used the Kaplan–Meier method to calculate survival estimates and time-to-event variables. Additionally, we compared the likelihood of returning from IFG to normoglycemia among the non-HDL-c/HDL-c ratio groups using the log-rank test.

To assess the possibility of covariate collinearity, we computed the variance inflation factor (VIF) [38]. The VIF was calculated using the formula VIF = 1/(1–R^2^), where R^2^ represented the R-squared value derived from a linear regression equation. For each regression analysis, the variable of interest served as the dependent variable, while all other variables were treated as independent variables. If the VIF exceeded 5, it signified collinearity among the variables and precluded their inclusion in the multiple regression model (Additional file 1: Table S1).

In order to explore the relationship between the non-HDL-c/HDL-c ratio and the probability of reverting to normoglycemia from IFG, three distinct models were constructed using univariate and multivariate Cox proportional-hazards regression analysis. The models employed in this study encompass three variations: Model I, which does not involve any adjustments to covariates; Model II, which includes adjustments solely for sociodemographic variables such as DBP, age, gender, family history of diabetes, SBP, smoking and drinking status, and BMI; and Model III, which incorporates adjustments for all covariates listed in Table 1, encompassing TG, gender, age, BUN, BMI, Scr, FPG, ALT, DBP, AST, family history of diabetes, SBP, LDL-c, smoking and drinking status. The study presented hazard ratios (HR) along with their corresponding 95% confidence intervals (CIs). The adjustment of confounding variables was based on clinical knowledge, published reports [27–29], and the results of collinearity screening, which indicated no collinearity issues among the variables (Additional file 1: Table S1).

**Table 1:** The baseline characteristics of participants

Non-HDL-c/HDL-c ratio	Q1 (< 2.19)	Q2 (2.19–2.67)	Q3 (2.67–3.44)	Q4 (≥ 3.45)	P-value
Participants	3803	3808	3807	3806	
Age (years)	48.6 ± 14.1	50.1 ± 13.3	52.5 ± 13.4	52.6 ± 12.6	< 0.001
BMI (kg/m^2^)	23.4 ± 3.3	24.8 ± 3.3	25.2 ± 3.1	25.8 ± 3.1	< 0.001
SBP (mmHg)	125 ± 18	127 ± 17	129 ± 18	129 ± 17	< 0.001
DBP (mmHg)	76 ± 11	78 ± 11	79 ± 11	80 ± 11	< 0.001
FPG (mmol/L)	5.9 ± 0.3	5.9 ± 0.3	6.0 ± 0.3	6.0 ± 0.3	< 0.001
TC (mmol/L)	4.4 ± 0.7	4.9 ± 0.8	5.2 ± 0.9	5.6 ± 0.9	< 0.001
TG (mmol/L)	0.9 (0.7–1.3)	1.3 (1.0–1.8)	1.6 (1.2–2.2)	2.1 (1.5–2.9)	< 0.001
HDL-c (mmol/L)	1.6 ± 0.3	1.4 ± 0.2	1.3 ± 0.2	1.1 ± 0.2	< 0.001
LDL-c (mmol/L)	2.5 ± 0.5	2.8 ± 0.6	3.1 ± 0.6	3.3 ± 0.8	< 0.001
Non-HDL-c (mmol/L)	2.9 ± 0.5	3.5 ± 0.6	3.9 ± 0.7	4.5 ± 0.8	< 0.001
Non-HDL-c/HDL-c	1.9 ± 0.3	2.4 ± 0.1	3.0 ± 0.2	4.24 ± 0.7	< 0.001
ALT (U/L)	18.0 (13.0–25.8)	21.0 (15.0–31.4)	23.0 (16.4–33.5)	26.6 (18.7–40.0)	< 0.001
AST (U/L)	24.3 ± 11.6	25.8 ± 12.0	26.7 ± 11.6	28.0 ± 11.7	< 0.001
BUN (mmol/L)	4.9 ± 1.2	5.0 ± 1.2	5.1 ± 1.3	5.0 ± 1.3	< 0.001
Scr (umol/L)	69.9 ± 15.4	73.3 ± 16.1	74.2 ± 16.8	74.4 ± 15.8	< 0.001
Gender					< 0.001
Male	2055 (54.0%)	2408 (63.2%)	2526 (66.4%)	2854 (75.0%)	
Female	1748 (46.0%)	1400 (36.8%)	1281 (33.6%)	952 (25.0%)	
Smoking status					< 0.001
Never smoker	3066 (80.6%)	2861 (75.1%)	2769 (72.7%)	2474 (65.0%)	
Ever smoker	141 (3.7%)	161 (4.2%)	149 (3.9%)	197 (5.2%)	
Current smoker	596 (15.7%)	786 (20.7%)	889 (23.4%)	1135 (29.8%)	
Drinking status					< 0.001
Never drinker	3105 (81.7%)	2969 (78.0%)	2992 (78.6%)	2962 (77.8%)	
Ever drinker	565 (14.8%)	680 (17.8%)	648 (17.0%)	655 (17.2%)	
Current drinker	133 (3.5%)	159 (4.2%)	167 (4.4%)	189 (5.0%)	
Family history of diabetes					0.205
No	3714 (97.7%)	3720 (97.7%)	3694 (97.0%)	3702 (97.3%)	
Yes	89 (2.3%)	88 (2.3%)	113 (3.0%)	104 (2.7%)	

Values are n (%), mean ± SD or medians (quartiles)

*BMI* body mass index, *LDL-c* low-density lipoprotein cholesterol, *FPG* fasting plasma glucose, *AST* aspartate aminotransferase, *DBP* diastolic blood pressure, *TC* total cholesterol, *Scr* serum creatinine; *SBP* systolic blood pressure; *BUN* blood urea nitrogen; *TG* triglyceride, *ALT* alanine aminotransferase; *HDL-c* high-density lipoprotein cholesterol, *non-HDL-c* non-high-density lipoprotein cholesterol, *non-HDL-c/HDL-c ratio* non-high-density lipoprotein cholesterol/high-density lipoprotein cholesterol ratio

To account for the potential non-linear correlation between the non-HDL-c/HDL-c ratio and reversion to normoglycemia in individuals with IFG, we employed a Cox proportional hazards regression model with cubic spline functions and the smooth curve fitting. This approach allowed us to address any non-linearity present in the data. When non-linearity was detected, we applied a recursive algorithm to identify the inflection point. Subsequently, we conducted two-piecewise Cox proportional hazards regression models on each side of the inflection point. To determine the most appropriate model for assessing the association between the non-HDL-c/HDL-c ratio and reversion to normoglycemia, we performed a log-likelihood ratio test [39].

Considering that persons who develop diabetes during the follow-up period are less likely to revert to normoglycemia, it is important to acknowledge that this could have an impact on detecting IFG reversal or altering the probability of such events [40, 41]. To address this issue, we implemented competing risks multivariate Cox proportional hazards regression, following the methodology described by Fine and Gray [41, 42]. In this approach, progression to diabetes was considered as a competing risk for the events of reversion to normoglycemia.

To perform subgroup analyses based on gender, SBP, age, BMI, TG, DBP, and FPG, we utilized a stratified Cox proportional hazards regression model. Firstly, for continuous variables such as age (< 45, ≥ 45 years), SBP (< 140, ≥ 140 mmHg), BMI (< 18.5, ≥ 18.5 to < 24, ≥ 24 to 28, ≥ 28 kg/m^2^), TG (< 1.7, ≥ 1.7 mmol/L), DBP (< 90, ≥ 90 mmHg), and FPG (< 6.1, ≥ 6.1 mmol/L), categorical variables were established using clinically significant cut-off points [43–46]. Secondly, each stratification was adjusted for all other factors, including the stratification factor itself (TG, gender, age, BUN, BMI, Scr, ALT, DBP, AST, FPG, family history of diabetes, SBP, LDL-c, smoking, and drinking status). Finally, we conducted a likelihood ratio test to evaluate interactions by comparing models with and without interaction terms [47, 48].

In order to ascertain the dependability of our results, a series of sensitivity analyses were performed. Initially, the non-HDL-c/HDL-c ratio was classified into quartiles and the P-value for the trend was evaluated to validate the outcomes obtained when treating it as a continuous variable. This approach also allowed us to explore potential non-linear relationships. It is worth noting that a family history of diabetes, smoking, and alcohol consumption has been strongly linked to an increased risk of developing diabetes [49, 50]. In our additional sensitivity analyses investigating the association between non-HDL-c/HDL-c ratio and reversion to normoglycemia in people with IFG, we excluded individuals with a family history of diabetes, smoking, or alcohol consumption. Moreover, due to incomplete data in approximately 70% of cases, we excluded drinking and smoking status as covariates in the multivariate model, as this might not contribute effectively to model adjustment. Moreover, a generalized additive model (GAM) was employed to incorporate the continuity covariate as a curve in model IV, aiming to ensure the consistency of the findings [51]. Additionally, E-values were computed to assess the potential influence of unmeasured confounding variables on the association between the non-HDL-c/HDL-c ratio and reversion to normoglycemia [52]. This methodological approach offered additional perspectives on the reliability of our outcomes.

The data analysis was performed utilizing two statistical software packages, namely R (The R Foundation, http://www.R-project.org) and EmpowerStats (X&Y Solutions, Inc, Boston, MA, http://www.empowerstats.com). All statistical tests were conducted as two-sided tests, and a significance level of P-value < 0.05 was employed to ascertain statistical significance.

## Results

### Baseline characteristics of participants

The baseline characteristics of the participants involved in the study are presented in Table 1. The mean age was 50.9 ± 13.5 years, with 64.7% being male. The baseline non-HDL-c/HDL-c ratio had a mean value of 2.9 ± 1.0. Among these individuals, 6357 (41.8%) with IFG achieved normoglycemia during a median follow-up period of 2.9 years. Participants were divided into subcategories based on quartiles of the non-HDL-c/HDL-c ratio (< 2.2, ≥ 2.2 to < 2.7, ≥ 2.7 to < 3.4, and ≥ 3.4). There were no significant differences in the family history of diabetes between the different quartiles of the non-HDL-c/HDL-c ratio (P values > 0.05). Comparing the Q4 (≥ 3.4) group with the Q1 (< 2.2) group, we observed significant increases in age, BMI, DBP, FPG, TC, SBP, TG, AST, LDL-c, BUN, non-HDL-c, ALT, Scr, male gender, ever and current smokers, and current drinkers. Conversely, there were opposite trends for HDL-c, females, never smokers, and never drinkers among the covariates.

**Fig. 2:**
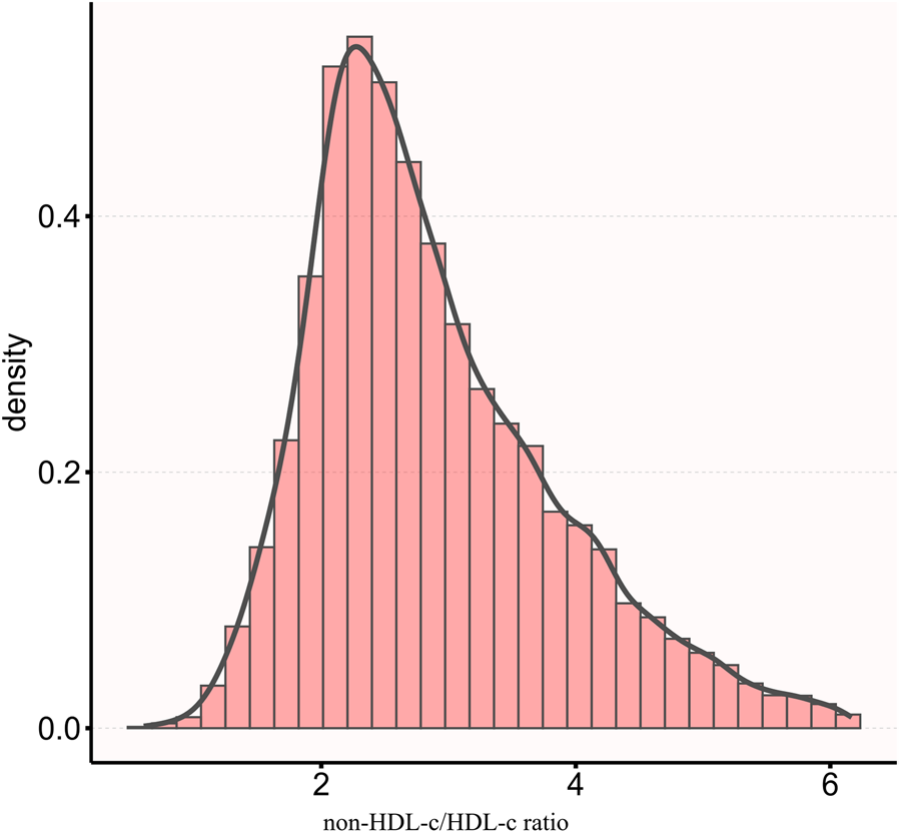
Distribution of non-HDL-c/HDL-c ratio. Presents a normal distribution of the non-HDL-c/HDL-c ratio, ranging from 0.6 to 6.2, and with a mean value of 2.9

**Fig. 3:**
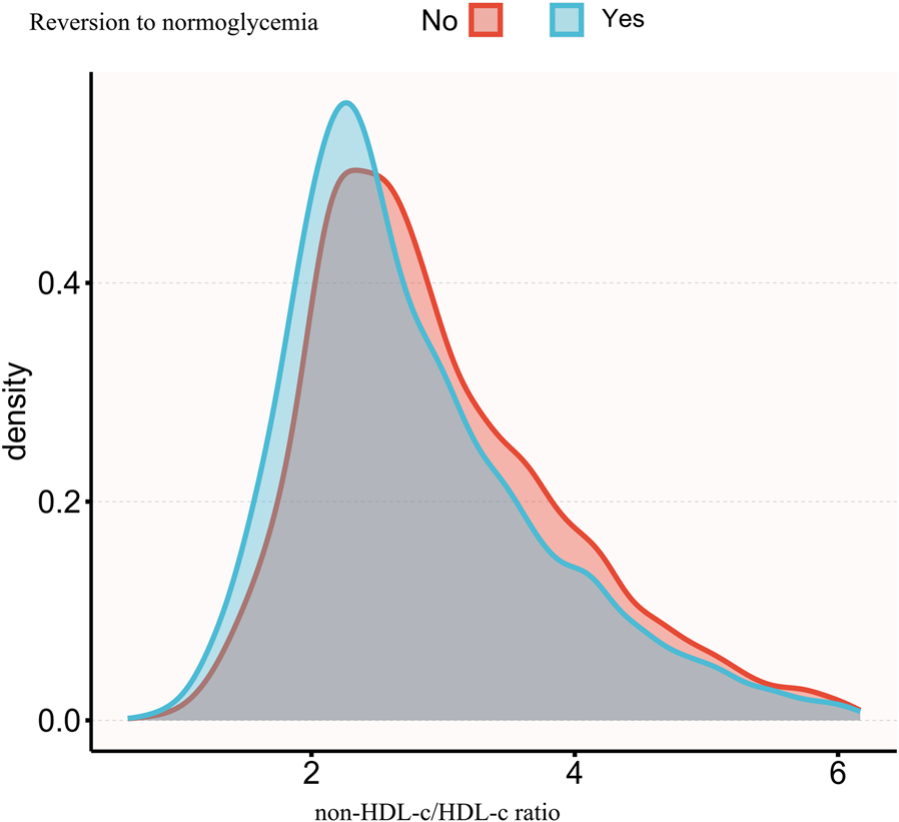
Data visualization of non-HDL-c/HDL-c ratio of all people with IFG who reverted to normoglycemia or not. Visually illustrates that people with IFG who reverted to normoglycemia experienced a notable reduction in the non-HDL-c/HDL-c ratio, in contrast to those who did not achieve normoglycemia

The normal distribution of the non-HDL-c/HDL-c ratio levels is illustrated in Fig. 2, with a range of values from 0.6 to 6.2, and a mean value of 2.9. Participants were classified into two distinct groups based on the occurrence of reversion to normoglycemia during the follow-up period. Figure 3 visually shows that individuals with IFG who achieved normoglycemia experienced a significant reduction in the non-HDL-c/HDL-c ratio compared to those who did not reach normoglycemia.

### The reversal rate to normoglycemia from IFG

**Fig. 4:**
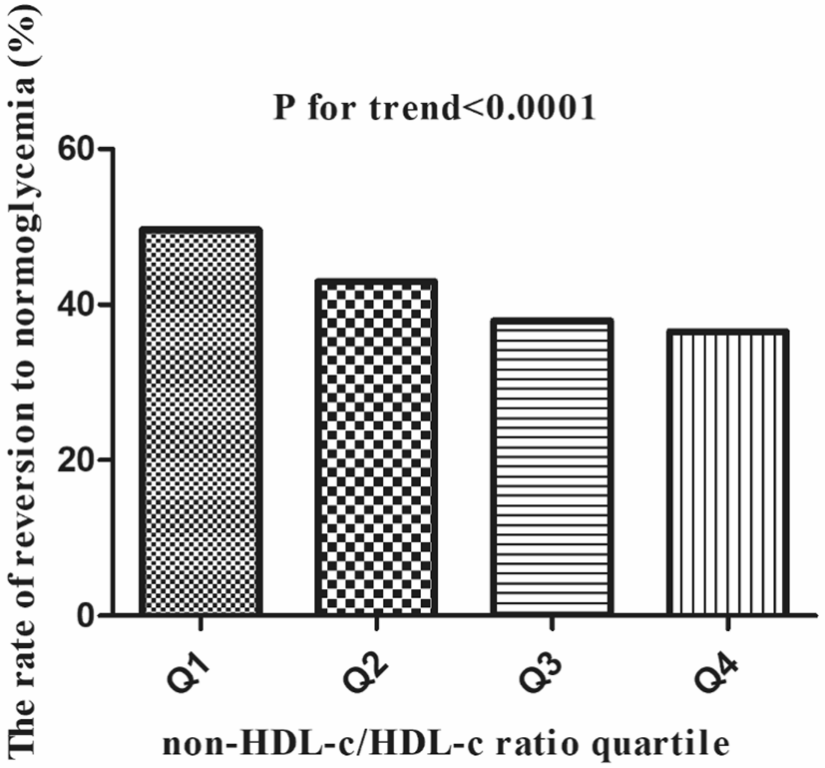
The rate of reversion to normoglycemia in people with IFG stratified by the quartiles of non-HDL-c/HDL-c ratio. Shows that participants with higher non-HDL-c/HDL-c ratio showed lower rates of reversal to normoglycemia (*P* < 0.001 for trend)

Among participants with IFG, a total of 6357 individuals achieved normoglycemia, resulting in an overall cumulative rate of 14.2 per 100 person-years. The cumulative rate of reversion to normoglycemia varied across the four non-HDL-c/HDL-c ratio groups for participants with IFG, ranging from 18.2 to 11.3 per 100 person-years. Specifically, the rates of reversion for the overall normoglycemia category and each non-HDL-c/HDL-c ratio group were as follows: 41.7% (41.0–42.5%), 49.6% (48.0–51.2%), 43.0% (41.4–44.6%), 37.9% (36.4–39.5%), and 36.5% (35.0–38.0%), respectively. It is worth noting that participants with higher non-HDL-c/HDL-c ratios demonstrated lower rates of reversion to normoglycemia (P < 0.0001 for trend). Further details can be found in Table 2 and Fig. 4.

**Table 2:** The rate of reversion to normoglycemia in people with IFG

Non-HDL-c/HDL-c ratio	Participants(n)	Reversion events(n)	Reversal rate (95% CI) (%)	Per 100 person-year
Total	15224	6357	41.8 (41.0–42.5)	14.2
Q1 (< 2.2)	3803	1887	49.6 (48.0–51.2)	18.2
Q2 (2.2–2.7)	3808	1637	43.0 (41.4–44.6)	15.4
Q3 (2.7–3.4)	3807	1444	37.9 (36.4–39.5)	12.6
Q4 (≥ 3.4)	3806	1389	36.5 (35.0–38.0)	11.3
P for trend			< 0.001	

*non-HDL-c/HDL-c ratio* non-high-density lipoprotein/high-density lipoprotein ratio, *CI* confidence interval

**Fig. 5:**
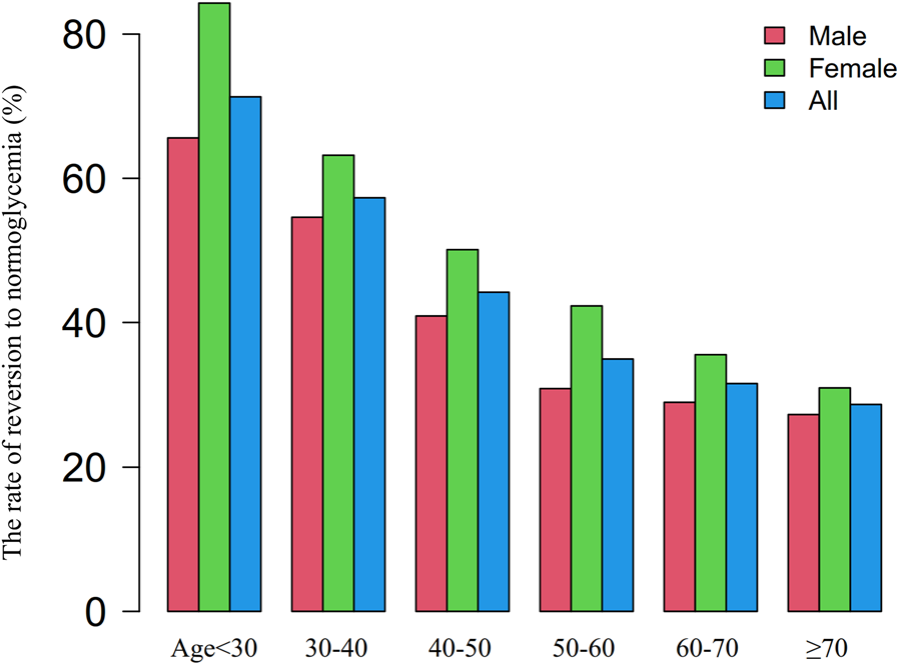
The rate of reversion to normoglycemia in people with IFG of age stratification by 10 intervals. According to Fig. 5, participants with IFG showed a higher rate of reversion to normoglycemia among women than men, regardless of their age group. Furthermore, the reversal rate in both men and women decreased with increasing age

Regarding age stratification in 10 year intervals, women exhibited higher reversion rates to normoglycemia than men across all age groups (Fig. 5). Moreover, both men and women experienced a decrease in the reversion rate as age increased.

### The results of univariate analyses using the Cox proportional-hazards regression model

The univariate analyses revealed that the reversion to normoglycemia was not associated with ever drinkers (P > 0.05), but it showed a positive correlation with being female and having higher levels of HDL-c. On the other hand, it was negatively associated with being male, older age, higher DBP, BMI, FPG, SBP, BUN, TG, Scr, LDL-c, ALT, non-HDL-c, non-HDL-c/HDL-c ratio, AST, a family history of diabetes, and being an ever or current smoker or current drinker (all P < 0.05; see Table 3).

**Table 3:** Factors influencing reversion to normoglycemia among participants with IFG analyzed by univariate Cox proportional hazards regression

Variable	Statistics	HR (95% CI)	P value
Age(years)	50.9 ± 13.5	0.97 (0.97, 0.98)	< 0.00001
Gender			
Male	9843 (64.7%)	Ref	
Female	5381 (35.3%)	1.25 (1.19, 1.31)	< 0.00001
BMI(kg/m^2^)	24.8 ± 3.3	0.93 (0.93, 0.94)	< 0.00001
SBP(mmHg)	127.5 ± 17.7	0.99 (0.99, 1.00)	< 0.00001
DBP(mmHg)	78.5 ± 11.2	0.98 (0.98, 0.99)	< 0.00001
FPG(mmol/L)	6.0 ± 0.3	0.21 (0.18, 0.22)	< 0.00001
TC(mmol/L)	5.0 ± 0.9	0.89 (0.86, 0.91)	< 0.00001
TG(mmol/L)	1.8 ± 1.4	0.90 (0.88, 0.92)	< 0.00001
HDL-c(mmol/L)	1.3 ± 0.3	1.97 (1.83, 2.13)	< 0.00001
LDL-c(mmol/L)	2.9 ± 0.7	0.94 (0.91, 0.98)	0.00069
Non-HDL-c (mmol/L)	3.7 ± 0.9	0.81 (0.78, 0.83)	< 0.00001
Non-HDL-c/HDL-c	2.9 ± 1.0	0.74 (0.72, 0.76)	< 0.00001
ALT(U/L)	28.0 ± 23.1	0.99 (0.99, 1.00)	< 0.00001
AST(U/L)	26.2 ± 11.8	0.99 (0.98, 0.99)	< 0.00001
BUN(mmol/L)	5.0 ± 1.2	0.96 (0.94, 0.98)	0.00001
Scr(umol/L)	73.0 ± 16.2	1.00 (1.00, 1.00)	0.00002
Smoking status			
Never smoker	11170 (73.4%)	Ref	
Ever smoker	648 (4.2%)	0.86 (0.76, 0.98)	0.02513
Current smoker	3406 (22.4%)	0.83 (0.78, 0.88)	< 0.00001
Drinking status			
Never drinker	12028 (79.0%)	Ref	
Ever drinker	2548 (16.7%)	0.98 (0.92, 1.05)	0.54647
Current drinker	648 (4.3%)	0.77 (0.67, 0.88)	0.00010
Family history of diabetes			
No	14830 (97.4%)	Ref	
Yes	394 (2.6%)	0.80 (0.68, 0.94)	0.00627

*BMI* body mass index, *FPG* fasting plasma glucose, *LDL-c* low-density lipoprotein cholesterol, *DBP* diastolic blood pressure, *ALT* alanine aminotransferase, *TC* total cholesterol, *SBP* systolic blood pressure, *TG* triglyceride, *AST* aspartate aminotransferase, *non-HDL-c* non-high-density lipoprotein cholesterol, *BUN* blood urea nitrogen, *HDL-c* high-density lipoprotein cholesterol, *Scr* serum creatinine, *non-HDL-c/HDL-c ratio* non-high-density lipoprotein cholesterol/high-density lipoprotein cholesterol ratio, *HR* Hazard ratios, *CI* confidence interval, *Ref* reference

**Fig. 6:**
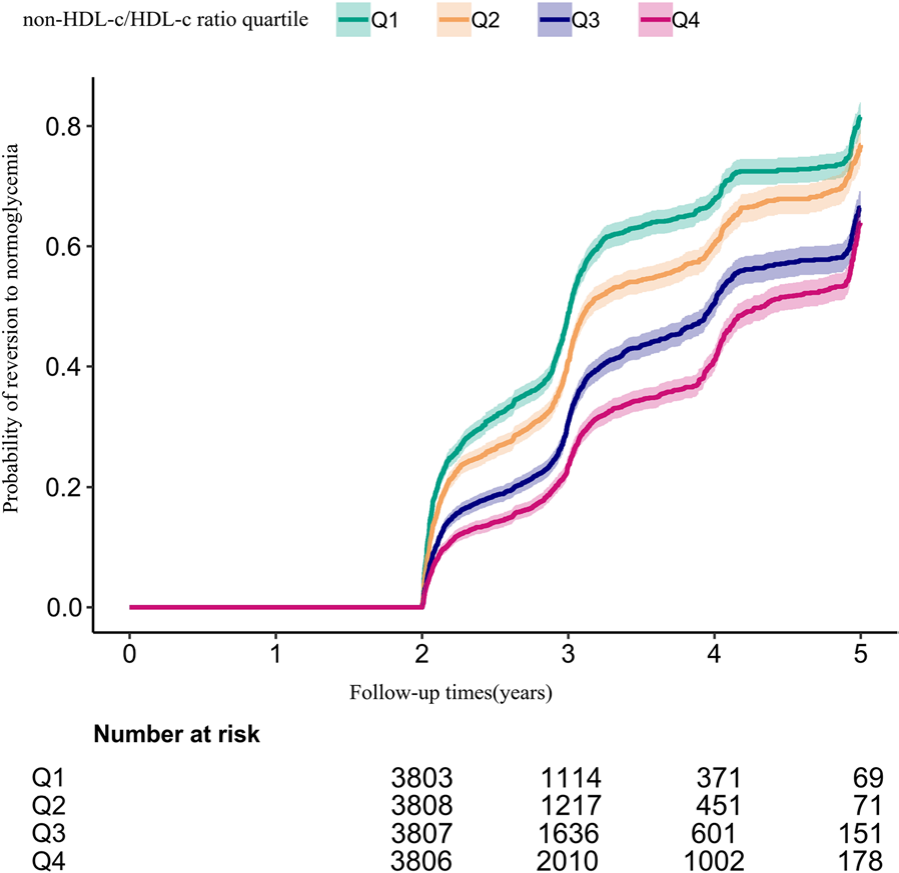
Kaplan–Meier curves for the probability of reversion to normoglycemia from IFG. Displays the Kaplan–Meier curves, illustrating the likelihood of reverting to normoglycemia from IFG, categorized by quartiles of the non-HDL-c/HDL-c ratio. The findings reveal a gradual decline in the probability of reversion to normoglycemia as the non-HDL-c/HDL-c ratio increases. This suggests that individuals with the highest non-HDL-c/HDL-c ratio have the lowest chance of transitioning from IFG to normoglycemia

Figure 6 presents the Kaplan–Meier curves illustrating the probability of reversion to normoglycemia from IFG across different categories of non-HDL-c/HDL-c ratios. The probability of reverting to normoglycemia significantly varied among the non-HDL-c/HDL-c ratio groups (log-rank test, p < 0.001). Notably, as the non-HDL-c/HDL-c ratio increased, the likelihood of reverting to normoglycemia progressively decreased. This suggests that individuals with higher non-HDL-c/HDL-c ratios had a lower probability of transitioning from IFG to normoglycemia.

### The results of multivariate analyses using the Cox proportional-hazards regression model

The authors utilized three Cox proportional-hazards regression models to investigate the association between the non-HDL-c/HDL-c ratio and the likelihood of reverting to normoglycemia. In the unadjusted model (Model I), a 1-unit increase in the non-HDL-c/HDL-c ratio was found to be significantly associated with a 26% decrease in the probability of returning to normoglycemia (HR = 0.74, 95% CI 0.72–0.76). The inclusion of confidence intervals in the results suggests that the observed relationship between the non-HDL-c/HDL-c ratio and the reversion to normoglycemia is statistically robust. In the minimally-adjusted model (Model II), adjusting solely for demographic variables, each additional unit of the non-HDL-c/HDL-c ratio was found to significantly decrease the likelihood of reversion to normoglycemia by 20% (HR = 0.80, 95% CI 0.78–0.82). The correlation between the non-HDL-c/HDL-c ratio and the reversion to normoglycemia from this model was statistically significant. In the fully-adjusted model (Model III), each additional unit of the non-HDL-c/HDL-c ratio was associated with a 29% decrease in the likelihood of reversion to normoglycemia (HR = 0.71, 95% CI 0.69–0.74). The results are statistically significant, as shown in Table 4.

**Table 4:** Relationship between non-HDL-c/HDL-c ratio and reversion to normoglycemia in people with IFG in different models

Exposure	Model I (HR, 95% CI, P)	Model II (HR, 95% CI, P)	Model III (HR, 95% CI, P)	Model IV (HR, 95% CI, P)
Non-HDL-c/HDL-c ratio	0.74 (0.72, 0.76) < 0.0001	0.80 (0.78, 0.82) < 0.0001	0.71 (0.69, 0.74) < 0.0001	0.70 (0.68, 0.73) < 0.0001
Non-HDL-c/HDL-c ratio				
Q1	Ref	Ref	Ref	Ref
Q2	0.81 (0.76, 0.87) < 0.0001	0.89 (0.84, 0.96) 0.0009	0.81 (0.76, 0.87) < 0.0001	0.80 (0.74, 0.85) < 0.0001
Q3	0.58 (0.54, 0.62) < 0.0001	0.68 (0.64, 0.74) < 0.0001	0.58 (0.54, 0.63) < 0.0001	0.56 (0.51, 0.60) < 0.0001
Q4	0.47 (0.44, 0.50) < 0.0001	0.57 (0.53, 0.61) < 0.0001	0.45 (0.41, 0.49) < 0.0001	0.43 (0.39, 0.48) < 0.0001
P for trend	< 0.0001	< 0.0001	< 0.0001	< 0.0001

Model I: we did not adjust other covariates

Model II: we adjust gender, age, BMI, SBP, DBP, family history of diabetes, smoking and drinking status

Model III: we adjust gender, age, BMI, SBP, DBP, FPG, BUN, Scr, TG, LDL-c, ALT, AST, family history of diabetes, smoking and drinking status

Model IV: we adjusted gender, age (smooth), BMI (smooth), SBP(smooth), Scr(smooth), DBP(smooth), FPG(smooth), BUN(smooth), TG(smooth), LDL-c(smooth), ALT(smooth), AST(smooth), family history of diabetes, smoking and drinking status

*HR* Hazard ratios, *CI* confidence, *Ref* reference

### The results of competing risks multivariate Cox proportional-hazards regression

Table 5 presents the findings of the competing analysis examining the progression from IFG to incident diabetes. In Model I (unadjusted), we observed a negative correlation between the non-HDL-c/HDL-c ratio and the likelihood of reverting to normoglycemia (SHR = 0.74, 95% CI 0.72–0.76). Model II (minimally-adjusted) was adjusted for various factors, including family history of diabetes, age, gender, SBP, BMI, DBP, smoking, and drinking status. The results of this model did not show any significant changes (SHR: 0.80, 95% CI 0.78–0.82). The fully adjusted model (Model III) took into account additional factors such as TG, BUN, FPG, ALT, AST, Scr, SBP, and LDL-c. This model also revealed a negative association between the non-HDL-c/HDL-c ratio and the reversion to normoglycemia (SHR = 0.71, 95% CI 0.69–0.74).

**Table 5:** Relationship between non-HDL-c/HDL-c ratio and reversion to normoglycemia in people with IFG in different models with competing risk of progression to diabetes

Exposure	Model I (SHR, 95% CI, P)	Model II (SHR, 95% CI, P)	Model III (SHR, 95% CI, P)
Non-HDL-c/HDL-c ratio	0.74 (0.72, 0.76) < 0.0001	0.80 (0.78, 0.82) < 0.0001	0.71 (0.69, 0.74) < 0.0001
Non-HDL-c/HDL-c ratio Quartile			
Q1	Ref	Ref	Ref
Q2	0.81 (0.76, 0.87) < 0.0001	0.89 (0.83, 0.95) 0.0009	0.81 (0.76, 0.87) < 0.0001
Q3	0.58 (0.54, 0.62) < 0.0001	0.68 (0.64, 0.73) < 0.0001	0.58 (0.54, 0.63) < 0.0001
Q4	0.47 (0.44, 0.50) < 0.0001	0.57 (0.53, 0.61) < 0.0001	0.45 (0.41, 0.49) < 0.0001
P for trend	< 0.0001	< 0.0001	< 0.0001

Model I: we did not adjust other covariates

Model II: we adjust gender, age, BMI, SBP, DBP, family history of diabetes, smoking and drinking status

Model III: we adjust gender, age, BMI, SBP, DBP, FPG, BUN, Scr, TG, LDL-c, ALT, AST, family history of diabetes, smoking and drinking status

*SHR* subdistribution hazard ratios, *CI* confidence, *Ref* reference

### Sensitivity analysis

In order to enhance the dependability of our results, we conducted a series of sensitivity analyses. Initially, we partitioned the non-HDL-c/HDL-c ratio into quartiles rather than treating it as a continuous variable. Subsequently, we reintegrated the non-HDL-c/HDL-c ratio, which had been transformed into categorical form, into the model. The findings indicated that following the transformation of the non-HDL-c/HDL-c ratio into categories, the effect sizes within each group displayed equidistant patterns, and the p-value for trend remained consistent with the outcomes obtained when the non-HDL-c/HDL-c ratio was treated as a continuous variable (refer to Tables 4, 5).

In order to incorporate the continuity covariate as a curve in the equation, a Generalized Additive Model (GAM) was utilized. The findings from Model IV in Table 4 were generally consistent with those obtained from the fully adjusted model, indicating an HR of 0.70 (95% CI 0.68–0.73, P < 0.0001). Additionally, E-values were computed to evaluate the sensitivity to unmeasured confounding variables. The resulting E-value of 1.85 suggests that any unmeasured or unknown confounders had minimal impact on the association between the non-HDL-c/HDL-c ratio and the probability of reverting to normoglycemia.

In addition, we conducted several supplementary sensitivity analyses to strengthen our findings. Specifically, we excluded individuals with a family history of diabetes or a history of smoking or alcohol consumption from our analysis. Remarkably, even after accounting for these confounding factors, we consistently observed a negative correlation between the non-HDL-c/HDL-c ratio and the probability of reverting to normoglycemia (Table 6).

**Table 6:** Relationship between non-HDL-c/HDL-c ratio and the probability of reverting from IFG to normoglycemia in different sensitivity analyses

Exposure	Model a (HR, 95% CI, P)	Model b (HR, 95% CI, P)	Model c (HR, 95% CI, P)	Model d (HR, 95% CI, P)
Non-HDL-c/HDL-c ratio	0.71 (0.68, 0.74) < 0.0001	0.71 (0.69, 0.74) < 0.0001	0.71 (0.69, 0.74) < 0.0001	0.71 (0.69, 0.74) < 0.0001
Non-HDL-c/HDL-c ratio (Quartile)				
Q1	Ref	Ref	Ref	Ref
Q2	0.81 (0.75, 0.87) < 0.0001	0.79 (0.73, 0.86) < 0.0001	0.81 (0.76, 0.87) < 0.0001	0.82 (0.76, 0.88) < 0.0001
Q3	0.57 (0.52, 0.62) < 0.0001	0.58 (0.53, 0.63) < 0.0001	0.58 (0.54, 0.63) < 0.0001	0.59 (0.54, 0.64) < 0.0001
Q4	0.45 (0.40, 0.50) < 0.0001	0.45 (0.41, 0.50) < 0.0001	0.45 (0.41, 0.49) < 0.0001	0.45 (0.41, 0.49) < 0.0001
P for trend	< 0.0001	< 0.0001	< 0.0001	< 0.0001

Model a was a sensitivity analysis performed on never smoker participants (N = 11170). We adjusted gender, age, BMI, SBP, DBP, FPG, BUN, Scr, TG, LDL-c, ALT, AST, family history of diabetes, and drinking status

Model b was a sensitivity analysis performed on never drinker participants (N = 12028). We adjusted gender, age, BMI, SBP, DBP, FPG, BUN, Scr, TG, LDL-c, ALT, AST, family history of diabetes, and smoking status

Model c was sensitivity analysis in participants without adjusting smoking and drinking status (N = 15224). We adjusted gender, age, BMI, SBP, DBP, FPG, BUN, Scr, TG, LDL-c, ALT, AST, family history of diabetes

Model d was sensitivity analysis in participants without family history of diabetes (N = 14830). We adjusted gender, age, BMI, SBP, DBP, FPG, BUN, Scr,TG, LDL-c, ALT, AST, smoking and drinking status

*HR* Hazard ratios, *CI* confidence, *Ref* reference

However, we encountered a significant challenge due to the substantial amount of missing data (approximately 70%) regarding smoking and alcohol consumption status. As a result, we had to exclude these variables as covariates in certain sensitivity analyses. Nevertheless, even without their inclusion, the findings remained consistent with our previous analyses (HR = 0.71, 95% CI 0.69–0.74) (refer to Table 6). These results from the sensitivity analysis further affirm the robustness of our findings.

### The non-linearity addressed by Cox proportional hazards regression model with cubic spline functions

**Fig. 7:**
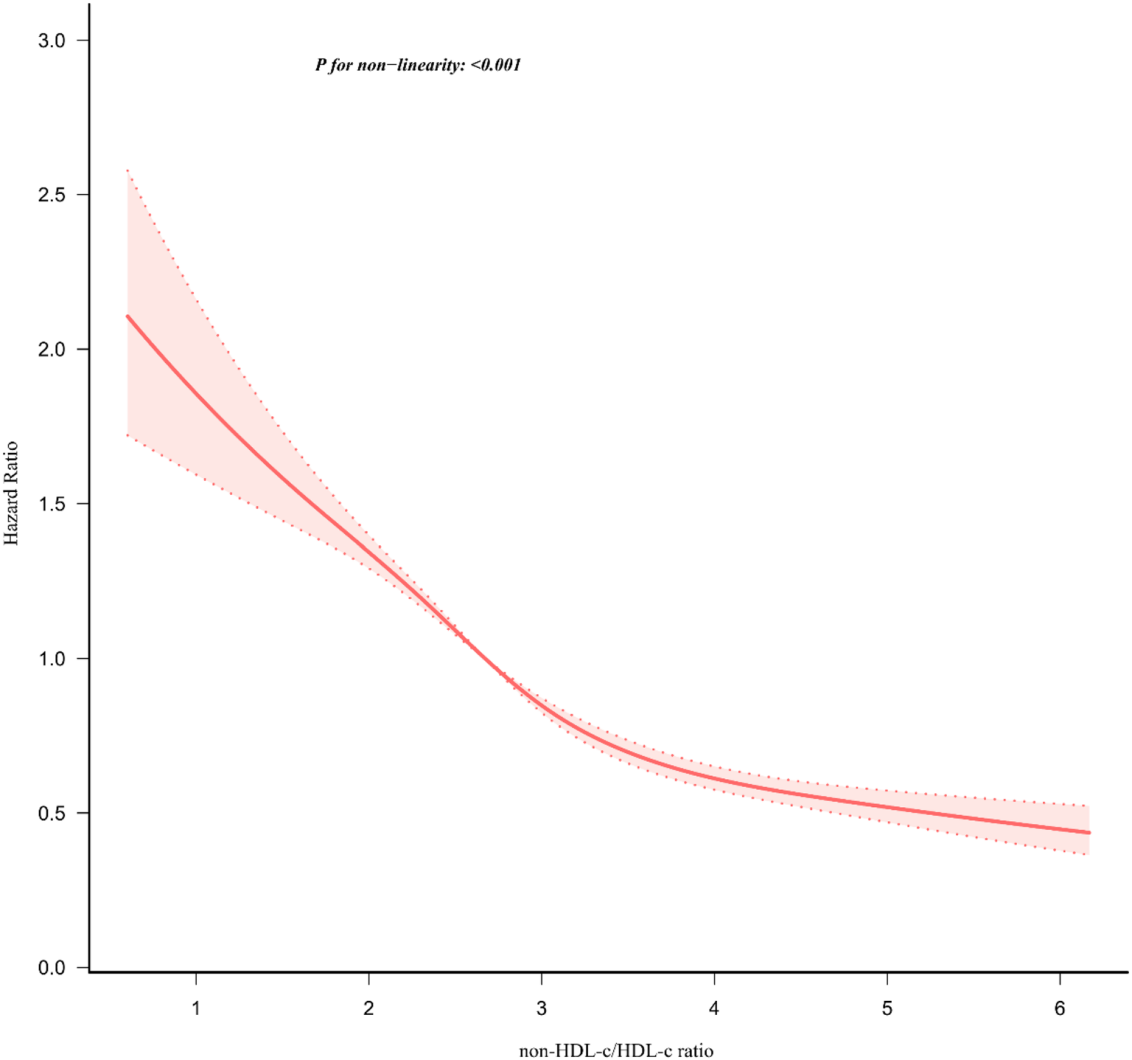
The non-linear relationship between the non-HDL-c/HDL-c ratio and reversion to normoglycemia in people with IFG. In Fig. 7, we employed a Cox proportional hazards regression model with cubic spline functions to investigate the association between the non-HDL-c/HDL-c ratio and the probability of reversion from IFG to normoglycemia. The findings reveal a non-linear relationship between the non-HDL-c/HDL-c ratio and this probability, with an inflection point observed at 3.1

Applying the Cox proportional hazards regression model with cubic spline functions, we made an intriguing observation that the association between the non-HDL-c/HDL-c ratio and the likelihood of reversion to normoglycemia from IFG is non-linear in nature (refer to Fig. 7). To delve deeper into this relationship, we employed a standard binary two-piecewise Cox proportional hazards regression model and the log-likelihood ratio test to determine the best fit. Remarkably, the P-value for this test was found to be below 0.05, indicating statistical significance (refer to Table 7).

**Table 7:** The result of the two-piecewise Cox regression model

Probability of reversion to normoglycemia	HR (95%CI)	P
Fitting model by standard Cox regression	0.71 (0.69, 0.74)	< 0.0001
Fitting model by two-piecewise Cox regression		
Inflection point of non-HDL-c/HDL-c ratio	3.1	
≤ Inflection point	0.63 (0.59, 0.67)	< 0.0001
> Inflection point	0.78 (0.74, 0.83)	< 0.0001
P for log-likelihood ratio test		< 0.001

We adjusted gender, age, BMI, SBP, DBP, FPG, BUN, Scr, TG, LDL-c, ALT, AST, family history of diabetes, smoking and drinking status

*HR*, Hazard ratios; *CI*: confidence, *Ref*: reference

By employing a recursive algorithm, we initially determined the inflection point to be 3.1, which facilitated the computation of hazard ratios (HR) and their corresponding confidence intervals (CIs) using the two-piecewise Cox proportional hazards regression model. On the left side of the inflection point, an HR of 0.63 (95% CI 0.59, 0.67) was observed, indicating a statistically significant effect. Conversely, on the right side of ction point, the effect size remained significant with an HR of 0.78 (95% CI 0.74, 0.83).

### The results of subgroup analyses

We comprehensively evaluated the interaction between various variables and the association between the non-HDL-c/HDL-c ratio and the likelihood of reverting to normoglycemia in both prespecified and exploratory subgroups (refer to Table 8). Our analysis indicated the absence of significant interactions concerning age, gender, BMI, SBP, DBP, or FPG. Nevertheless, a notable interaction was observed with the variable TG.

**Table 8:** Stratified associations between non-HDL-c/HDL-c ratio and reversion to normoglycemia in people with IFG in prespecified and exploratory subgroups

Characteristic	No of participants	HR (95%CI)	P value	P for interaction
Age(years)		0.70 (0.67, 0.74)		0.5084
< 45	5258	0.70 (0.67, 0.74)	< 0.0001	
≥ 45	9966	0.72 (0.69, 0.75	< 0.0001	
Gender				0.5175
Male	9843	0.71 (0.68, 0.74)	< 0.0001	
Female	5381	0.72 (0.68, 0.76)	< 0.0001	
BMI (kg/m^2^)				0.5877
< 18.5	257	0.76 (0.57, 1.01)	0.0590	
≥ 18.5, < 24	5972	0.69 (0.65, 0.73)	< 0.0001	
≥ 24, < 28	6575	0.71 (0.67, 0.75)	< 0.0001	
≥ 28	2420	0.74 (0.67, 0.80)	< 0.0001	
SBP (mmHg)				
< 140	11809	0.71 (0.68, 0.73)	< 0.0001	
DBP (mmHg)				
< 90	12969	0.71 (0.68, 0.73)	< 0.0001	
≥ 90	2255	0.73 (0.67, 0.79)	< 0.0001	
TG (mmol/L)				
< 1.7	9292	0.68 (0.65, 0.72)	< 0.0001	
≥ 1.7	5932	0.76 (0.73, 0.80)	< 0.0001	
FPG (mmol/L)				0.2422
< 6.1	11060	0.71 (0.69, 0.74)		< 0.0001
≥ 6.1	4164	0.68 (0.63, 0.73)		< 0.0001

Note 1: Above model adjusted for gender, age, BMI, SBP, DBP, FPG, BUN, Scr, TG, LDL-c, ALT, AST, family history of diabetes, smoking and drinking status

Note 2: In each case, the model is not adjusted for the stratification variable

*HR* Hazard ratios, *CI* confidence, *Ref* reference

Specifically, we found a more pronounced association between the non-HDL-c/HDL-c ratio and the probability of reverting to normoglycemia among participants with TG levels less than 1.7 mmol/L (HR = 0.68, 95% CI 0.65–0.72). In contrast, the association between the non-HDL-c/HDL-c ratio and the likelihood of reversion to normoglycemia from IFG was attenuated among participants with TG levels greater than 1.7 mmol/L (HR = 0.76, 95% CI 0.73–0.80).

## Discussion

In this retrospective cohort study, we investigated the relationship between the non-HDL-c/HDL-c ratio and the likelihood of reversion to normoglycemia in individuals with IFG. Our findings revealed a significant association between increasing non-HDL-c/HDL-c ratios and a decreased probability of reversion to normoglycemia. Furthermore, we identified a threshold effect curve, which indicated varying correlations between the non-HDL-c/HDL-c ratio and reversion to normoglycemia on either side of the inflection point. Notably, we also found that TG levels acted as potential effect modifiers, influencing the correlation between the non-HDL-c/HDL-c ratio and reversion to normoglycemia. Specifically, significantly stronger associations were observed among individuals with TG levels below 1.7 mmol/L, whereas significantly weaker associations were detected among those with TG levels greater than 1.7 mmol/L.

The findings of a prospective cohort study, which included a total of 491 participants, revealed that 22.6% of people with IFG experienced a return to normoglycemia over a median follow-up time of 2.5 years [53]. Another study reported a return to normoglycemia in 54% of participants after 1 year, while 6% developed diabetes [54]. Likewise, a cohort research conducted in China involving a sample size of 14,231 adults demonstrated that during a span of 2 years, 44.9% of those diagnosed with IFG experienced a reversion to normoglycemia [14]. In our study, which spanned a 5 year period, we observed a reversal to normoglycemia in 41.8% of the cases. These variations in the rates of reversion across studies may be attributed to discrepancies in participant age, duration of follow-up, and ethnic background. Nevertheless, all studies confirm that a substantial proportion of individuals with IFG have the potential to revert to normoglycemia. Therefore, it is crucial to identify the contributing factors to this reversion process in order to prevent diabetes and its associated complications.

A cohort study conducted in rural China demonstrated that the risk of incident T2DM was significantly higher in quartiles 2, 3, and 4 compared to quartile 1 of the baseline non-HDL-c/HDL-c ratio. The HR for quartiles 2, 3, and 4 were 1.46 (95% CI 1.08–1.98), 1.51 (95% CI 1.12–2.03), and 2.16 (95% CI 1.62–2.88), respectively. Additionally, an increase in the non-HDL-c/HDL-c ratio during the follow-up period was associated with a higher risk of T2DM. The HR for quartile 3 was 1.67 (95% CI 1.25–2.24), and for quartile 4, it was 2.00 (95% CI 1.52–2.61) [55]. Similarly, another 3 year cohort study in China demonstrated that each standard deviation increase in log10 non-HDL-c/HDL-c ratio was associated with a higher risk of diabetes (OR 1.10; 95% CI 1.00–1.20) in a multivariate model [32]. Furthermore, a cohort study conducted on Japan's general population revealed an 18% increase in the likelihood of developing diabetes (HR 1.18, 95% CI 1.07–1.30) for every 1 unit rise in the non-HDL-c/HDL-c ratio. Moreover, the utilization of ROC curve analysis demonstrated that the non-HDL-c/HDL-c ratio outperformed conventional lipid parameters as a more effective indicator for predicting the risk of diabetes [27]. Considering that the progression to diabetes mellitus and the reversal to normoglycemia represent opposite developments in individuals with IFG, it was hypothesized that an increased non-HDL-c/HDL-c ratio might be associated with a lower probability of regression to normoglycemia from IFG. However, no previous studies have explored this relationship. Our present study has now confirmed that an elevated non-HDL-c/HDL-c ratio is indeed linked to a lower probability of reversal to normoglycemia in people with IFG. Furthermore, this negative association remained consistent even after excluding smoking and drinking status from the multiple regression equation. Sensitivity analyses demonstrated a consistent relationship among participants without a family history of diabetes, non-smokers, and non-alcohol consumers. Prior studies have demonstrated that a short period of achieving normal blood glucose levels considerably decreases the likelihood of developing T2DM in those with IFG [11]. Therefore, the objective of treating IFG should mainly focus on restoring normoglycemia rather than solely preventing its progression to diabetes mellitus. The findings from our study have significant clinical implications, suggesting that clinicians should consider interventions to decrease non-HDL-c/HDL-c ratio levels to promote regression to normoglycemia from IFG.

The exact mechanism by which a decrease in the non-HDL-c/HDL-c ratio contributes to the promotion of regression to normoglycemia from IFG remains unclear. However, previous research has indicated that the accumulation of excess cholesterol can lead to β-cell dysfunction, resulting in impaired glucose tolerance and compromised insulin secretion. Furthermore, deposition of cholesterol in the islets may contribute to increased aggregation of islet amyloid polypeptide and the formation of islet amyloid, further exacerbating β-cell dysfunction and impacting glucose regulation [23–26, 56]. Therefore, it is plausible that the reduction in non-HDL-c/HDL-c ratio levels may have improved β-cell function, ultimately facilitating the reversal of IFG to normoglycemia.

Based on our current understanding, our research represents the initial investigation into the presence of a non-linear correlation between the non-HDL-c/HDL-c ratio and the return to normoglycemia among individuals with IFG. In order to elucidate this non-linear association, we utilized a two-piecewise Cox proportional hazards regression model and determined an inflection point at 3.1, while accounting for potential confounding variables. Our findings revealed that as the non-HDL-c/HDL-c ratio decreased below 3.1, the probability of reversion to normoglycemia increased by 37% for each unit decrease in the non-HDL-c/HDL-c ratio (HR = 0.63, 95% CI 0.59–0.67). Conversely, when the non-HDL-c/HDL-c ratio exceeded 3.1, a decrease in the ratio by 1 unit was associated with a 22% increase in the probability of reversing to normoglycemia (HR = 0.78, 95% CI 0.74–0.83). Therefore, a decrease in non-HDL-c/HDL-c ratio levels in individuals with IFG is linked to an increased likelihood of reversion to normoglycemia. However, it is worth noting that the rate of increase in the probability of returning to normoglycemia was found to be accelerated when the non-HDL-c/HDL-c ratio was below 3.1. In contrast, it was observed to be decelerated when the ratio surpassed 3.1.

The rationale for considering other variables in participants' baseline is their potential impact on diabetes risk. Comparing individuals with a non-HDL-c/HDL-c ratio < 3.1 to those with a ratio ≥ 3.1, it was observed that the latter group generally had higher levels of age, FPG, BMI, Scr, TG, ALT, LDL-c, SBP, AST, DBP, current smokers, and drinkers (Additional file 1: Table S2). However, it is important to note that these indicators are closely associated with diabetes [24, 30, 49, 57–60]. When the non-HDL-c/HDL-c ratio exceeds 3.1, the presence of these diabetes risk factors diminishes the impact of the non-HDL-c/HDL-c ratio on diabetes risk. Conversely, when the ratio is below 3.1, the levels of diabetes risk factors such as BMI, Scr, FPG, TG, ALT, LDL-c, SBP, and DBP tend to be lower, resulting in a weakened effect on diabetes. Therefore, at this point, the influence of the non-HDL-c/HDL-c ratio on diabetes risk relatively increases. These findings provide a crucial rationale for intervention in the non-HDL-c/HDL-c ratio level within clinical settings to increase the likelihood of reversing to normoglycemia. Importantly, the observation of a negative association between the probability of reversing to normoglycemia and a non-HDL-c/HDL-c ratio below 3.1 is noteworthy. This study offers valuable insights into strategies for increasing the probability of reversing to normoglycemia for individuals with different non-HDL-c/HDL-c ratio statuses. From a therapeutic standpoint, it is recommended to keep the levels of non-HDL-c/HDL-c ratio below the inflection point. In the present condition, a reduction in the ratio can substantially impact the likelihood of returning to a state of normal blood glucose levels. Consequently, this study holds significant clinical significance. The findings of this research are expected to contribute to future efforts aimed at establishing predictive models for assessing the probability of reversing normoglycemia from IFG.

In the analysis of subgroups, it was observed that TG could potentially influence the association between the non-HDL-c/HDL-c ratio and the probability of returning to normoglycemia, with more pronounced associations observed in individuals with TG levels below 1.7 mmol/L. Conversely, significantly weaker associations were found in the population with TG levels exceeding 1.7 mmol/L. In conjunction with prior investigations, this study has provided further evidence that TG levels exceeding 1.7 mmol/L are strongly associated with an elevated risk of developing diabetes [24, 61]. Hence, it is unsurprising that the correlation between the non-HDL-c/HDL-c ratio and the restoration of normal blood glucose levels is attenuated in the subgroup of individuals with TG levels greater than 1.7 mmol/L, as TG exerts an influential effect on this particular outcome. Given that TG can modify the relationship between the non-HDL-c/HDL-c ratio and the likelihood of reversion to normoglycemia, there is a clinical possibility to increase the probability of reversion by altering the strength of this association through interventions targeting TG levels. In clinical practice, we can enhance the probability of reversion to normoglycemia by reducing non-HDL-c/HDL-c ratio levels and simultaneously increase reversion effectiveness by further lowering TG levels.

Our study has several notable strengths, which are listed below. Firstly, it is the first study to specifically investigate the relationship between the non-HDL-c/HDL-c ratio and reversion to normoglycemia from IFG in the Chinese population. This unique focus provides valuable insights into this particular population. Secondly, identifying a non-linear connection between the non-HDL-c/HDL-c ratio and restoring normoglycemia from IFG, along with determining inflection points, substantially enhances the current understanding in this domain. Thirdly, to tackle missing data, we employed a multiple imputation technique to optimize statistical power and minimize bias arising from incomplete covariate information. The inclusion of this methodological rigor significantly enhances the dependability of our outcomes. Fourthly, we performed a sequence of sensitivity analyses to further substantiate the resilience of our discoveries. These additional analyses strengthen the credibility of the observed associations. Furthermore, we employed a multivariate Cox proportional hazards regression model, accounting for the competing risk of IFG progressing to diabetes, in assessing the likelihood of reversion to normoglycemia. This comprehensive approach provides a more nuanced understanding of the factors influencing reversion outcomes.

The study has several limitations that should be taken into account. Firstly, the exclusive inclusion of Chinese participants in this study necessitates further investigation to ascertain the correlation between the non-HDL-c/HDL-c ratio and the restoration of normoglycemia in individuals with IFG from diverse genetic backgrounds. Secondly, while IFG serves as an indicator of pre-diabetes, it does not fully capture the complexity of the condition. Although measuring 2 h oral glucose tolerance tests and HbA1C levels in such a large study cohort was challenging, we aim to address this limitation in future studies or collaborate with other researchers to collect this valuable information. Thirdly, due to the utilization of a secondary analysis of published data, the ability to account for variables not encompassed within the original dataset, such as insulin concentration and waist circumference, was unattainable. Nonetheless, through the computation of the E-value, it was ascertained that the impact of unmeasured confounders on the outcomes was improbable. Fourthly, as this investigation was conducted post hoc and based on observational data, it established an association between the non-HDL-c/HDL-c ratio and regression of normoglycemia in people with IFG rather than a causal relationship. Fifthly, this study solely examined the non-HDL-c/HDL-c ratio and other parameters at the initial stage, neglecting their temporal fluctuations. In forthcoming research endeavors, we intend to design our studies or establish partnerships with fellow scholars to collect multiple data points, encompassing longitudinal data on the dynamic alterations of the non-HDL-c/HDL-c ratio throughout participant's follow-up. Finally, it is known that many people whose initial glucose value is within the range of IFG will return to normal in a repeat measurement. Some reasons are: (1) random variation, especially if the initial value is near the lower border; and regression to the mean phenomenon. Indeed, there was a strong inverse association between regression to normal and FPG indicating these issues.

## Conclusion

This study presents empirical evidence of a significant inverse correlation between the non-HDL-c/HDL-c ratio and the likelihood of reverting to normoglycemia in Chinese adults diagnosed with IFG. Furthermore, the study reveals a non-linear relationship and a discernible threshold effect between the non-HDL-c/HDL-c ratio and the achievement of normoglycemic status. These discoveries provide valuable insights for enhancing the prospects of transitioning from IFG to normoglycemia in individuals with varying non-HDL-c/HDL-c ratio statuses in the future. Notably, when the non-HDL-c/HDL-c ratio falls below 3.1, a noteworthy reduction in the ratio significantly enhances the likelihood of achieving normoglycemia. From a therapeutic perspective, it is advisable to maintain non-HDL-c/HDL-c ratio levels below the inflection point identified in this study.

### Supplementary Information

**Additional file 1: Table S1.** Collinearity diagnostics steps. **Table S2.** The characteristics of participants on both sides of the inflection point.

## Data Availability

The ‘DATADRYAD’ database (https://datadryad.org/stash) offers access to the data.

## Abbreviations

IDF: International diabetes federation
Non-HDL-c/HDL-c ratio: Non-high-density lipoprotein to high-density lipoprotein ratio
DPPOS: Diabetes prevention program outcomes study
CKD: Chronic kidney disease
Non-HDL-c: Non-high-density lipoprotein cholesterol
CVD: Chronic cardiovascular disease
T2DM: Type 2 diabetes mellitus
SBP: Systolic blood pressure
NAFLD: Nonalcoholic fatty liver disease
Scr: Serum creatinine
HbA1c: Glycated hemoglobin A1c
ALT: Alanine aminotransferase
DBP: Diastolic blood pressure
TG: Triglyceride
BMI: Body mass index
AST: Aspartate aminotransferase
LDL-c: Low-density lipid cholesterol
VIF: Variance inflation factor
BUN: Blood urea nitrogen
FPG: Fasting plasma glucose
HDL-c: High-density lipoprotein cholesterol
GAM: Generalized additive models
HR: Hazard ratios
TC: Otal cholesterol
IFG: Impaired fasting glucose
Ref: Reference
MAR: Missing-at-random
CI: Confidence intervals
SD: Standard deviation
IGT: Impaired glucose tolerance

## References

[1] Schmidt AM (2018). Highlighting diabetes mellitus: the epidemic continues. Arterioscl Throm Vas.

[2] Khetan AK, Rajagopalan S (2018). Prediabetes. Can J Cardiol.

[3] 2. Classification and Diagnosis of Diabetes: Standards of Medical Care in Diabetes-2022. Diabetes Care 2022, 45(Suppl 1):S17-S38 10.2337/dc22-S002 34964875

[4] Saeedi P, Petersohn I, Salpea P, Malanda B, Karuranga S, Unwin N, Colagiuri S, Guariguata L, Motala AA, Ogurtsova K (2019). Global and regional diabetes prevalence estimates for 2019 and projections for 2030 and 2045: results from the international diabetes federation diabetes Atlas, 9(th) edition. Diabetes Res Clin Pr.

[5] Forouhi NG, Luan J, Hennings S, Wareham NJ (2007). Incidence of type 2 diabetes in England and its association with baseline impaired fasting glucose: the ely study 1990–2000. Diabetic Med.

[6] Nathan DM, Davidson MB, DeFronzo RA, Heine RJ, Henry RR, Pratley R, Zinman B (2007). Impaired fasting glucose and impaired glucose tolerance: implications for care. Diabetes Care.

[7] Tabák AG, Herder C, Rathmann W, Brunner EJ, Kivimäki M (2012). Prediabetes: a high-risk state for diabetes development. Lancet.

[8] Ford ES, Zhao G, Li C (2010). Pre-diabetes and the risk for cardiovascular disease: a systematic review of the evidence. J Am Coll Cardiol.

[9] Warren B, Pankow JS, Matsushita K, Punjabi NM, Daya NR, Grams M, Woodward M, Selvin E (2017). Comparative prognostic performance of definitions of prediabetes: a prospective cohort analysis of the atherosclerosis risk in communities (ARIC) study. Lancet Diabetes Endo.

[10] Huang Y, Cai X, Mai W, Li M, Hu Y (2016). Association between prediabetes and risk of cardiovascular disease and all cause mortality: systematic review and meta-analysis. BMJ-Brit Med J.

[11] Perreault L, Pan Q, Mather KJ, Watson KE, Hamman RF, Kahn SE (2012). Effect of regression from prediabetes to normal glucose regulation on long-term reduction in diabetes risk: results from the diabetes prevention program outcomes study. Lancet.

[12] Pratte KA, Johnson A, Beals J, Bullock A, Manson SM, Jiang L (2019). Regression to normal glucose regulation in American Indians and Alaska natives of a diabetes prevention program. Diabetes Care.

[13] Perreault L, Temprosa M, Mather KJ, Horton E, Kitabchi A, Larkin M, Montez MG, Thayer D, Orchard TJ, Hamman RF (2014). Regression from prediabetes to normal glucose regulation is associated with reduction in cardiovascular risk: results from the diabetes prevention program outcomes study. Diabetes Care.

[14] Liu X, Wu S, Song Q, Wang X (2021). Reversion from pre-diabetes mellitus to normoglycemia and risk of cardiovascular disease and all-cause mortality in a Chinese population: a prospective cohort study. J Am Heart Assoc.

[15] Taskinen MR (2003). Diabetic dyslipidaemia: from basic research to clinical practice. Diabetologia.

[16] Ley SH, Harris SB, Connelly PW, Mamakeesick M, Gittelsohn J, Wolever TM, Hegele RA, Zinman B, Hanley AJ (2012). Utility of non-high-density lipoprotein cholesterol in assessing incident type 2 diabetes risk. Diabetes Obes Metab.

[17] Brunham LR, Kruit JK, Hayden MR, Verchere CB (2010). Cholesterol in beta-cell dysfunction: the emerging connection between HDL cholesterol and type 2 diabetes. Curr Diabetes Rep.

[18] Seo MH, Bae JC, Park SE, Rhee EJ, Park CY, Oh KW, Park SW, Kim SW, Lee WY (2011). Association of lipid and lipoprotein profiles with future development of type 2 diabetes in nondiabetic Korean subjects: a 4-year retrospective, longitudinal study. J Clin Endocr Metab.

[19] Zhu L, Lu Z, Zhu L, Ouyang X, Yang Y, He W, Feng Y, Yi F, Song Y (2015). Lipoprotein ratios are better than conventional lipid parameters in predicting coronary heart disease in Chinese Han people. Kardiol Pol.

[20] Wang D, Wang L, Wang Z, Chen S, Ni Y, Jiang D (2018). Higher non-HDL-cholesterol to HDL-cholesterol ratio linked with increased nonalcoholic steatohepatitis. Lipids Health Dis.

[21] Zuo PY, Chen XL, Liu YW, Zhang R, He XX, Liu CY (2015). Non-HDL-cholesterol to HDL-cholesterol ratio as an independent risk factor for the development of chronic kidney disease. Nutr Metab Cardiovas.

[22] Kim SW, Jee JH, Kim HJ, Jin SM, Suh S, Bae JC, Kim SW, Chung JH, Min YK, Lee MS (2013). Non-HDL-cholesterol/HDL-cholesterol is a better predictor of metabolic syndrome and insulin resistance than apolipoprotein B/apolipoprotein A1. Int J Cardiol.

[23] Ye Y, Gao J, Liang J, Yang Y, Lv C, Chen M, Wang J, Zhu D, Rong R, Xu M (2021). Association between preoperative lipid profiles and new-onset diabetes after transplantation in Chinese kidney transplant recipients: a retrospective cohort study. J Clin Lab Anal.

[24] Peng J, Zhao F, Yang X, Pan X, Xin J, Wu M, Peng YG (2021). Association between dyslipidemia and risk of type 2 diabetes mellitus in middle-aged and older Chinese adults: a secondary analysis of a nationwide cohort. BMJ Open.

[25] Bai Z, Zhang DS, Zhang R, Yin C, Wang RN, Huang WY, Ding J, Yang JL, Huang PY, Liu N (2021). A nested case-control study on relationship of traditional and combined lipid metabolism indexes with incidence of diabetes. Zhonghua Liu Xing Bing Xue Za Zhi.

[26] Yang T, Liu Y, Li L, Zheng Y, Wang Y, Su J, Yang R, Luo M, Yu C (2022). Correlation between the triglyceride-to-high-density lipoprotein cholesterol ratio and other unconventional lipid parameters with the risk of prediabetes and type 2 diabetes in patients with coronary heart disease: a RCSCD-TCM study in China. Cardiovasc Diabetol.

[27] Sheng G, Liu D, Kuang M, Zhong Y, Zhang S, Zou Y (2022). Utility of non-high-density lipoprotein cholesterol to high-density lipoprotein cholesterol ratio in evaluating incident diabetes risk. Diabet Metab Synd Ob.

[28] Busquets-Cortés C, Bennasar-Veny M, López-González ÁA, Fresneda S, Abbate M, Yáñez AM (2021). Utility of fatty liver index to predict reversion to normoglycemia in people with prediabetes. PLoS ONE.

[29] Han Y, Hu H, Huang Z, Liu D (2023). Association between body mass index and reversion to normoglycemia from impaired fasting glucose among Chinese adults: a 5-year cohort study. Front Endocrinol.

[30] Chen Y, Zhang XP, Yuan J, Cai B, Wang XL, Wu XL, Zhang YH, Zhang XY, Yin T, Zhu XH (2018). Association of body mass index and age with incident diabetes in Chinese adults: a population-based cohort study. BMJ Open.

[31] Geleris J, Sun Y, Platt J, Zucker J, Baldwin M, Hripcsak G, Labella A, Manson DK, Kubin C, Barr RG (2020). Observational study of hydroxychloroquine in hospitalized patients with covid-19. New Engl J Med.

[32] Zhang N, Hu X, Zhang Q, Bai P, Cai M, Zeng TS, Zhang JY, Tian SH, Min J, Huang HT (2018). Non-high-density lipoprotein cholesterol: high-density lipoprotein cholesterol ratio is an independent risk factor for diabetes mellitus: results from a population-based cohort study. J Diabetes.

[33] Chen Y, Zhang X, Pan B, Jin X, Yao H, Chen B, Zou Y, Ge J, Chen H (2010). A modified formula for calculating low-density lipoprotein cholesterol values. Lipids Health Dis.

[34] Lazo-Porras M, Bernabe-Ortiz A, Ruiz-Alejos A, Smeeth L, Gilman RH, Checkley W, Málaga G, Miranda JJ (2020). Regression from prediabetes to normal glucose levels is more frequent than progression towards diabetes: the CRONICAS cohort study. Diabetes Res Clin Pr.

[35] Hwang YC, Cho IJ, Jeong IK, Ahn KJ, Chung HY (2018). Factors associated with regression from prediabetes to normal glucose tolerance in a Korean general population: a community-based 10-year prospective cohort study. Diabetic Med.

[36] Groenwold RH, White IR, Donders AR, Carpenter JR, Altman DG, Moons KG (2012). Missing covariate data in clinical research: when and when not to use the missing-indicator method for analysis. Can Med Assoc J.

[37] White IR, Royston P, Wood AM (2011). Multiple imputation using chained equations: issues and guidance for practice. Stat Med.

[38] Wax Y (1992). Collinearity diagnosis for a relative risk regression analysis: an application to assessment of diet-cancer relationship in epidemiological studies. Stat Med.

[39] Rothenbacher D, Rehm M, Iacoviello L, Costanzo S, Tunstall-Pedoe H, Belch J, Söderberg S, Hultdin J, Salomaa V, Jousilahti P (2020). Contribution of cystatin C- and creatinine-based definitions of chronic kidney disease to cardiovascular risk assessment in 20 population-based and 3 disease cohorts: the BiomarCaRE project. BMC Med.

[40] Noordzij M, Leffondré K, van Stralen KJ, Zoccali C, Dekker FW, Jager KJ (2013). When do we need competing risks methods for survival analysis in nephrology?. Nephrol Dial Transpl.

[41] Basak R, Mistry H, Chen RC (2021). Understanding competing risks. Int J Radiat Oncol.

[42] Solbak NM, Al RA, Akawung AK, Lo SG, Kirkpatrick SI, Robson PJ (2019). Strategies to address misestimation of energy intake based on self-report dietary consumption in examining associations between dietary patterns and cancer risk. Nutrients.

[43] Hemmingsen B, Sonne DP, Metzendorf MI, Richter B (2017). Dipeptidyl-peptidase (DPP)-4 inhibitors and glucagon-like peptide (GLP)-1 analogues for prevention or delay of type 2 diabetes mellitus and its associated complications in people at increased risk for the development of type 2 diabetes mellitus. Cochrane Db Syst Rev.

[44] Chen Z, Hu H, Chen M, Luo X, Yao W, Liang Q, Yang F, Wang X (2020). Association of Triglyceride to high-density lipoprotein cholesterol ratio and incident of diabetes mellitus: a secondary retrospective analysis based on a Chinese cohort study. Lipids Health Dis.

[45] Karpov Y, Khomitskaya Y (2015). PROMETHEUS: an observational, cross-sectional, retrospective study of hypertriglyceridemia in Russia. Cardiovasc Diabetol.

[46] Pop-Busui R, Stevens MJ, Raffel DM, White EA, Mehta M, Plunkett CD, Brown MB, Feldman EL (2013). Effects of triple antioxidant therapy on measures of cardiovascular autonomic neuropathy and on myocardial blood flow in type 1 diabetes: a randomised controlled trial. Diabetologia.

[47] Mullee A, Romaguera D, Pearson-Stuttard J, Viallon V, Stepien M, Freisling H, Fagherazzi G, Mancini FR, Boutron-Ruault MC, Kühn T (2019). Association between soft drink consumption and mortality in 10 European Countries. Jama Intern Med.

[48] Keidel D, Anto JM, Basagaña X, Bono R, Burte E, Carsin AE, Forsberg B, Fuertes E, Galobardes B, Heinrich J (2019). The role of socioeconomic status in the association of lung function and air pollution-a pooled analysis of three adult ESCAPE cohorts. Int J Env Res Pub He.

[49] Sun Y, Ni W, Yuan X, Chi H, Xu J (2020). Prevalence, treatment, control of type 2 diabetes and the risk factors among elderly people in Shenzhen: results from the urban Chinese population. BMC Public Health.

[50] Schleger F, Linder K, Walter L, Heni M, Brändle J, Brucker S, Pauluschke-Fröhlich J, Weiss M, Häring HU, Preissl H (2018). Family history of diabetes is associated with delayed fetal postprandial brain activity. Front Endocrinol.

[51] Zhu F, Chen C, Zhang Y, Chen S, Huang X, Li J, Wang Y, Liu X, Deng G, Gao J (2020). Elevated blood mercury level has a non-linear association with infertility in U.S. women: data from the NHANES 2013–2016. Reprod Toxicol.

[52] Haneuse S, VanderWeele TJ, Arterburn D (2019). Using the E-value to assess the potential effect of unmeasured confounding in observational studies. JAMA-J Am Med Assoc.

[53] Sevilla-González M, Merino J, Moreno-Macias H, Rojas-Martínez R, Gómez-Velasco DV, Manning AK (2021). Clinical and metabolomic predictors of regression to normoglycemia in a population at intermediate cardiometabolic risk. Cardiovasc Diabetol.

[54] Bodicoat DH, Khunti K, Srinivasan BT, Mostafa S, Gray LJ, Davies MJ, Webb DR (2017). Incident type 2 diabetes and the effect of early regression to normoglycaemia in a population with impaired glucose regulation. Diabetic Med.

[55] Han M, Li Q, Qie R, Guo C, Zhou Q, Tian G, Huang S, Wu X, Ren Y, Zhao Y (2020). Association of non-HDL-C/HDL-C ratio and its dynamic changes with incident type 2 diabetes mellitus: the rural Chinese cohort study. J Diabetes Complicat.

[56] Ouchi G, Komiya I, Taira S, Wakugami T, Ohya Y (2022). Triglyceride/low-density-lipoprotein cholesterol ratio is the most valuable predictor for increased small, dense LDL in type 2 diabetes patients. Lipids Health Dis.

[57] Mo Z, Hu H, Du X, Huang Q, Chen P, Lai L, Yu Z (2021). Association of evaluated glomerular filtration rate and incident diabetes mellitus: a secondary retrospective analysis based on a Chinese cohort study. Front Med-Lausanne.

[58] Wu Y, Hu H, Cai J, Chen R, Zuo X, Cheng H, Yan D (2022). Association of mean arterial pressure with 5-year risk of incident diabetes in Chinese adults: a secondary population-based cohort study. BMJ Open.

[59] Porter SA, Pedley A, Massaro JM, Vasan RS, Hoffmann U, Fox CS (2013). Aminotransferase levels are associated with cardiometabolic risk above and beyond visceral fat and insulin resistance: the framingham heart study. Arterioscl Throm Vas.

[60] Tirosh A, Shai I, Tekes-Manova D, Israeli E, Pereg D, Shochat T, Kochba I, Rudich A (2005). Normal fasting plasma glucose levels and type 2 diabetes in young men. New Engl J Med.

[61] Mooradian AD (2009). Dyslipidemia in type 2 diabetes mellitus. Nat Clin Pract Endocrinol Metab.

