# Supplementary material for: Association between non-high-density lipoprotein to high-density lipoprotein ratio and reversion to normoglycemia in people with impaired fasting glucose: a 5-year retrospective cohort study
Source: Diabetol Metab Syndr. 2023 Dec 17;15:259. doi: 10.1186/s13098-023-01237-0 (PMC10726583; doi:10.1186/s13098-023-01237-0)
Supplement: Supplementary file 1 — Additional file 1: Table S1. Collinearity diagnostics steps. Table S2. The characteristics of participants on both sides of the inflection point. [file 13098_2023_1237_MOESM1_ESM.docx]

**Association between non-high-density lipoprotein to high-density lipoprotein ratio and reversion to normoglycemia in people with impaired fasting glucose: a 5-year retrospective cohort study**

**Running title：**Non-HDL-c/HDL-c ratio and reversion to normoglycemia

**Zihe Mo^1#^, Yong Han^2,3#^, Changchun Cao^4#^, Qingli Huang^1^, Yanhua Hu^5*^, Zhiqun Yu^1*^, Haofei Hu^6,7*^**

^1^Department of Physical Examination, DongGuan Tungwah Hospital, Dongguan 523000, Guangdong Province, China

^2^Department of Emergency, Shenzhen Second People’s Hospital, Shenzhen 518000, Guangdong Province, China

^3^Department of Emergency, The First Affiliated Hospital of Shenzhen University, Shenzhen 518000, Guangdong Province, China

^4^Department of Rehabilitation, Shenzhen Dapeng New District Nan’ao People’s Hospital, Shenzhen 518000, Guangdong Province, China

^5^College of Information Science and Engineering, Liuzhou Institute of Technology, Liuzhou 545616,

Guangxi Zhuang Autonomous Region, China

^6^Department of Nephrology, Shenzhen Second People’s Hospital, Shenzhen 518000, Guangdong Province, China

^7^Department of Nephrology, The First Affiliated Hospital of Shenzhen University, Shenzhen 518000, Guangdong Province, China

**# Zihe Mo, Yong Han, and Changchun Cao have contributed equally to this work.**

***Corresponding author**

Yanhua Hu,

College of Information Science and Engineering,

Liuzhou Institute of Technology,

No. 99, Xinliu Avenue, Yufeng District

Liuzhou 545616,

Guangxi Zhuang Autonomous Region,

China

E-mail: 19418601@qq.com

***Corresponding author**

Zhiqun Yu

Department of Physical Examination, DongGuan Tungwah Hospital,

No. 1 Dongcheng Road, Dongcheng Street,

Dongguan 523000,

Guangdong Province,

China

E-mail: yuzhiqun12345@126.com

***Corresponding author**

**Haofei Hu**

Department of Nephrology,

Shenzhen Second People’s Hospital,

No.3002 Sungang Road, Futian District,

Shenzhen 518000,

Guangdong Province,

China

E-mail: [huhaofei0319@126.com](mailto:huhaofei0319@126.com)

**Table S1. Collinearity diagnostics steps.**

| Variable | VIF  Step 1 |
| --- | --- |
|  |  |
| Gender | 2.1 |
| Age(years) | 1.4 |
| Smoking status | 1.3 |
| Drinking status | 1.2 |
| Family history of diabetes | 1.0 |
| LDL-c(mmol/L) | 1.0 |
| BUN(mmol/L) | 1.2 |
| FPG(mmol/L) | 1.1 |
| ALT(U/L) | 3.6 |
| AST(U/L) | 3.2 |
| TG (mmol/L) | 1.1 |
| Scr (umol/L) | 1.8 |
| BMI (kg/m^2^) | 1.3 |
| SBP (mmHg) | 1.9 |
| DBP (mmHg) | 2.8 |

BMI, body mass index; FPG, fasting plasma glucose; DBP, diastolic blood pressure; TC, total cholesterol; SBP, systolic blood pressure; TG, triglyceride; ALT, alanine aminotransferase; LDL-c, low-density lipoprotein cholesterol; AST, aspartate aminotransferase; HDL-c, high-density lipoprotein cholesterol; BUN, blood urea nitrogen; Scr, serum creatinine.

Abbreviation: VIF: variance inflation factor; VIF = 1/(1-R^2^).

Note: The variables with VIF>5 will be regarded as collinear variables and cannot be included in the multiple regression model.

**Table S2. The characteristics of participants on both sides of the inflection point.**

| Non-HDL-c/HDL-c ratio | <3.1 | >=3.1 | P-value |
| --- | --- | --- | --- |
| Participants | 9878 | 5346 |  |
| Age (years) | 50.0 ± 13.7 | 52.7 ± 12.8 | <0.001 |
| BMI (kg/m^2^) | 24.3 ± 3.3 | 25.7 ± 3.1 | <0.001 |
| SBP (mmHg) | 127 ± 18 | 129 ± 17 | <0.001 |
| DBP (mmHg) | 78 ± 11 | 80 ± 11 | <0.001 |
| FPG (mmol/L) | 5.9 ± 0.3 | 6.0 ± 0.3 | <0.001 |
| TC (mmol/L) | 4.8 ± 0.8 | 5.51 ± 0.91 | <0.001 |
| TG (mmol/L) | 1.2(0.9-1.7) | 2.0 (1.4-2.8) | <0.001 |
| HDL-c (mmol/L) | 1.5 ± 0.3 | 1.1 ± 0.2 | <0.001 |
| LDL-c (mmol/L) | 2.7 ± 0.6 | 3.3 ± 0.8 | <0.001 |
| Non-HDL-c (mmol/L) | 3.3 ± 0.7 | 4.4 ± 0.8 | <0.001 |
| ALT (U/L) | 20.1 (14.3-29.8) | 25.5 (18.0-38.0) | <0.001 |
| AST (U/L) | 25.4 ± 11.7 | 27.7 ± 11.8 | <0.001 |
| BUN (mmol/L) | 5.0 ± 1.2 | 5.0 ± 1.3 | 0.004 |
| Scr (umol/L) | 72.3 ± 16.1 | 74.1 ± 16.2 | <0.001 |
| Gender |  |  | <0.001 |
| Male | 5959 (60.3%) | 3884 (72.4%) |  |
| Female | 3919 (39.7%) | 1462 (27.3%) |  |
| Smoking status |  |  | <0.001 |
| Never smoker | 7597 (76.9%) | 3573 (66.8%) |  |
| Ever smoker | 384 (3.9%) | 264 (4.9%) |  |
| Current smoker | 1897 (19.2%) | 1509 (28.3%) |  |
| Drinking status |  |  | 0.012 |
| Never drinker | 7847 (79.4%) | 4181 (78.2%) |  |
| Ever drinker | 1645 (16.7%) | 903 (16.9%) |  |
| Current drinker | 386 (3.9%) | 262 (4.9%) |  |
| Family history of diabetes |  |  | 0.075 |
| No | 9639 (97.6%) | 5191 (97.1%) |  |
| Yes | 239 (2.4%) | 155 (2.9%) |  |

Values are n (%), mean±SD or medians (quartiles)

BMI, body mass index; FPG, fasting plasma glucose; DBP, diastolic blood pressure; TC, total cholesterol; SBP, systolic blood pressure; TG, triglyceride; ALT, alanine aminotransferase; LDL-c, low-density lipoprotein cholesterol; AST, aspartate aminotransferase; HDL-c, high-density lipoprotein cholesterol; non-HDL-c, non-high-density lipoprotein cholesterol; BUN, blood urea nitrogen; Scr, serum creatinine.
